# Visual and kinesthetic modes affect motor imagery classification in untrained subjects

**DOI:** 10.1038/s41598-019-46310-9

**Published:** 2019-07-08

**Authors:** Parth Chholak, Guiomar Niso, Vladimir A. Maksimenko, Semen A. Kurkin, Nikita S. Frolov, Elena N. Pitsik, Alexander E. Hramov, Alexander N. Pisarchik

**Affiliations:** 1 Center for Biomedical Technology, Technical University of Madrid, Pozuelo de Alarcón, 28223 Madrid, Spain; 2 Innopolis University, Innopolis, 420500 Russia; 3 Biomedical Image Technologies, Technical University of Madrid and CIBER-BBN, Madrid, Spain

**Keywords:** Image processing, Personality, Brain-machine interface, Scientific data

## Abstract

The understanding of neurophysiological mechanisms responsible for motor imagery (MI) is essential for the development of brain-computer interfaces (BCI) and bioprosthetics. Our magnetoencephalographic (MEG) experiments with voluntary participants confirm the existence of two types of motor imagery, kinesthetic imagery (KI) and visual imagery (VI), distinguished by activation and inhibition of different brain areas in motor-related *α*- and *β*-frequency regions. Although the brain activity corresponding to MI is usually observed in specially trained subjects or athletes, we show that it is also possible to identify particular features of MI in untrained subjects. Similar to real movement, KI implies muscular sensation when performing an imaginary moving action that leads to event-related desynchronization (ERD) of motor-associated brain rhythms. By contrast, VI refers to visualization of the corresponding action that results in event-related synchronization (ERS) of *α*- and *β*-wave activity. A notable difference between KI and VI groups occurs in the frontal brain area. In particular, the analysis of evoked responses shows that in all KI subjects the activity in the frontal cortex is suppressed during MI, while in the VI subjects the frontal cortex is always active. The accuracy in classification of left-arm and right-arm MI using artificial intelligence is similar for KI and VI. Since untrained subjects usually demonstrate the VI imagery mode, the possibility to increase the accuracy for VI is in demand for BCIs. The application of artificial neural networks allows us to classify MI in raising right and left arms with average accuracy of 70% for both KI and VI using appropriate filtration of input signals. The same average accuracy is achieved by optimizing MEG channels and reducing their number to only 13.

## Introduction

The revealing features of electrical and magnetic brain activity during imagination of the movement of different limbs are very important for fundamental neuroscience and various applications, such as brain-computer interfaces (BCIs) which can help in rehabilitation of patients after trauma or stroke, as well as for noninvasive brain-controlled bioprostheses and exoskeletons^1–3^. Mental imagination of movements referred to as *motor imagery* (MI)^4^ manifests as a result of the rehearsal of a given motor act in the working memory without any overt movement of the corresponding muscle. It is classified into two categories, namely, visual imagery (VI) and kinesthetic imagery (KI). While VI consists of visualization of the subject moving a limb, that does not require any special training or sensing of the muscles, KI is the feeling of muscle movement, that can usually be achieved by athletes or specially trained persons^5^.

MI has been studied using different experimental techniques (for a comprehensive review see Guillot *et al*.^6^). The most popular techniques are functional magnetic resonance imaging (fMRI)^7,8^, positron emission tomography (PET)^9–11^, electroencephalography (EEG)^12–17^, transcranial magnetic stimulation (TMS)^5,18–21^, and magnetoencephalography (MEG)^22–26^. Previous studies using fMRI^7,8^ indicate that brain activity associated with KI is similar to real movement because it includes commands of muscle contraction which are then blocked at some level of the motor system by inhibitory mechanisms. This enables MI to activate a large part of the same neuronal network which is involved in the real movement, but without any movement of the corresponding muscle during the motor imagination. Diverse TMS studies^5,18–21^ also confirm the overlapping activity in the brain areas during KI and real movement.

To understand and classify MI, many methods of time-frequency and spatio-temporal analyses are used. Among them, the most common techniques are using event-related synchronization (ERS) and event-related desynchronization (ERD)^22,27–29^, power spectral density, wavelet transform, empirical mode decomposition, common spatial patterns, spatio-decomposition, as well as their combinations^30–32^. In addition, for classification of brain states associated with MI, the methods of machine learning and artificial intelligence are also applied to analyze EEG and MEG time series^33–35^.

Although in the majority of papers devoted to MI the EEG approach was used, there was extensive research using MEG^22–26^. The advantages of MEG over EEG is that MEG provides a higher spatial resolution and less susceptibility to artifacts. However, to the best of our knowledge, the MEG experiments with untrained subjects were not carried out. Nonetheless, several researchers studied the effects of cognitive training on memory performance^36^ and visual detection^37^ in healthy subjects and subjects with pathologies^38,39^, as well as musical training in healthy musicians and non-musicians^40–42^. In particular, a relatively good accuracy was achieved in classification between left-hand and right-hand MI and between MI and a rest state using the combination of a spatio-spectral decomposition and a common spatial patterns analysis^25^. Furthermore, both MEG and EEG were used in brain-computer interfaces for training MI classifiers^26^. The authors demonstrated rather efficient classification of MI even without separation of participants into KI and VI categories. At the same time, it was shown that KI and VI scenarios affect the classification accuracy, e.g., the accuracy rate obtained for KI were better than for VI^16^. In this context, taking into account that untrained subjects often demonstrate the VI imagery mode, the possibility to increase the accuracy rate for VI is in demand for BCI applications.

With this in mind, the aim of the present work is twofold: (i) to understand the cognitive brain behavior associated with MI by conducting MEG experiments with untrained subjects and (ii) to obtain information about imagery-related brain activity for developing optimal strategies which would provide maximal accuracy rate in classification between left-arm and right-arm MI in both groups of subjects.

## Materials and Methods

### Participants

**Figure 1:**
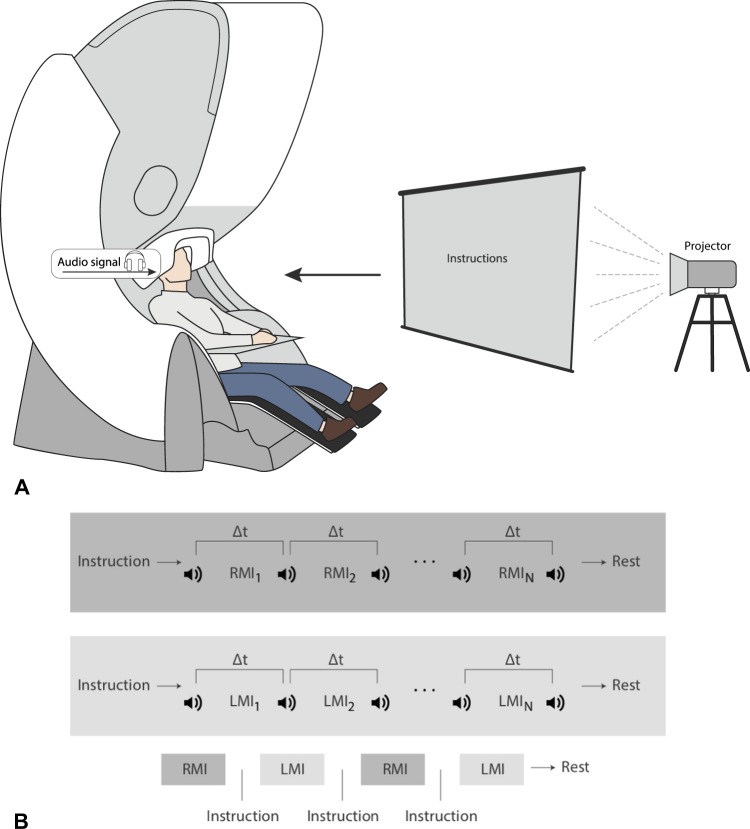
Design of the MEG experiment on motor imagery (MI). (**A**) Schematic representation of experimental performance and (**B**) experimental algorithm. RMI _*i*_ and LMI _*i*_ are time intervals corresponding to right-arm and left-arm MI, respectively, *i* indicates the subsequent trial number, and Δ*t* denotes the duration of each trial.

The experimental study consisted of ten untrained volunteers, eight males and two females between the ages of 20 and 31. The subjects were sat in a comfortable reclining chair with their legs straight and arms resting on an armrest in front of them, as shown in Fig. 1. All participants were required to imagine moving their arms after being presented with audible beeps as the cue.

#### Ethics statement

All participants provided a written informed consent before the experiment commencement. The experimental studies were performed in accordance with the Declaration of Helsinki. Methods were carried out in accordance with approved guidelines. The research was approved by the Ethics Committee of the Technical University of Madrid.

### Experimental equipment

The neurophysiological data were acquired with the Vectorview MEG system (Elekta AB, Stockholm, Sweden) with 306 channels (102 magnetometers and 204 planar gradiometers) placed inside a magnetically shielded room (Vacuum Schmelze GmbH, Hanau, Germany) at the Laboratory of Cognitive and Computational Neuroscience, Center for Biomedical Technology, Technical University of Madrid, Spain. Fastrak digitizer (Polhemus, Colchester, Vermont) was used to obtain the three-dimensional head shape. Three fiducial points (nasion, left and right preauricular) and more than 300 points on the scalp were acquired for each subject. Vertical electrooculogram (EOG) was placed to capture blinks and other undesirable eye movements. The sampling frequency was 1000 Hz and an online anti-alias bandpass filter between 0.1 and 330 Hz was utilized.

### Experimental procedure

The whole MEG experiment was divided into four series with one-fourth of the total number of trials in each series (Fig. 1). Each series consisted of equal number of trials randomly chosen for each of two arms (left or right arm MI). Before changing the arm, the subject was informed on the screen about which arm he/she must imagine to move after the beep. Each beep followed the subject imagining to move the arm. The imaginary movement of each arm was counted as one trial. The beeps were presented with time gaps randomly varied from 6 to 8 seconds. The number of trials per limb was varied among the subjects from 16 to 28. We provided a 20-s gap after finishing all trials for each arm and a resting 60-s interval between each series. The reason for incorporating multiple trials for the same limb continuously, as opposed to single trials sparsely presented, was to obtain longer uninterrupted data, free of transients and more task-focussed.

### Data acquisition and pre-processing

The MATLAB code was used to produce all audio and visual commands in this study to log the time of the beginning and end of each movement imagination (event) in a protocol file. This text file was then used to find the moments of time corresponding to the beginning and end of each imaginary movement when analyzing the MEG file (in .fif format) using the Brainstorm interface^43^. Artifacts in the MEG recordings were removed using the temporal signal-space separation method of Taulu and Hari^44^. Once the events were marked at the beginning of each limb movement imagination, we extracted the 5-s trials just after these marks. Similarly, the 20-s trials corresponding to the resting state with closed eyes were also marked as the background activity of each subject.

### Time-frequency analysis

The time-frequency structure of the MEG signals was analyzed using wavelet-based approach, a well-known tool for the analysis of non-stationary time series in biology and medicine^45^. For each limb, we used the Morlet wavelet with *f*_0_ = 1 Hz central frequency and a 3-s full width at half maximum (FWHM) to evaluate the time-frequency spectrogram (TFS) for all extracted epochs, and then averaged the TFSs for the limb. Then, the TFS was also averaged over the desired motor-related frequency ranges of *α* (8–12 Hz) and *β* (15–30 Hz) bands. The same process was repeated over the background resting state using the same parameters. To evaluate ERS/ERD, we took the difference between the spectrogram for the trials and the averaged-over-time spectrogram of the background and then normalized it to the background (resting state). For ERS/ERD, this normalized difference yielded positive/negative values.

The wavelet energy *E*^*n*^(*f*, *t*) = |*W*_*n*_(*f*, *t*)|^2^ was calculated for every MEG channel *X*_*n*_(*t*) for particular frequency *f*. Here, *W*_*n*_(*f*, *t*) is a complex-valued wavelet coefficient numerically calculated as^46^

1 $${W}_{n}(f,t)=\sqrt{f}{\int }_{t-4/f}^{t+4/f}\,{X}_{n}(t){\psi }^{\ast }(f,t)dt,$$

where *n* = 1, …, *N* is the channel number (*N* = 102 being the total number of MEG magnetometers) and “*” defines complex conjugation. The mother wavelet function *ψ*(*f*, *t*) is the Morlet wavelet often used for the analysis of neurophysiological data, defined as

2 $$\psi (f,t)=\sqrt{f}{\pi }^{1/4}{{\rm{e}}}^{j{\omega }_{0}f(t-{t}_{0})}{{\rm{e}}}^{f{(t-{t}_{0})}^{2}/2},$$

where *ω*_0_ = 2*πf*_0_ = 2*π* is the central frequency of the mother Morlet wavelet.

The values of the wavelet energy ${E}_{\alpha }^{n}(t)$ and ${E}_{\beta }^{n}(t)$ were calculated, respectively, in the alpha (8–12 Hz) and beta (15–30 Hz) frequency bands for each *n*-th MEG channel as

3 $${E}_{\alpha ,\beta }^{n}(t)=\frac{1}{{\rm{\Delta }}f}\mathop{\int }\limits_{f\in \alpha ,\beta }\,{E}^{n}(f,t)df.$$

The event-related fields $L{A}_{\alpha }^{n}(t)$ and $L{A}_{\beta }^{n}(t)$, $R{A}_{\alpha }^{n}(t)$ and $R{A}_{\beta }^{n}(t)$, $B{C}_{\alpha }^{n}(t)$ and $B{C}_{\beta }^{n}(t)$ were extracted for each limb by averaging the values ${E}_{\alpha }^{n}(t)$ and ${E}_{\beta }^{n}(t)$ over the trials corresponding to left-arm (LA) and right-arm (RA) MI, and background activity (BC), respectively.

Finally, in order to estimate ERD (or ERS) associated with left-arm and right-arm MI, we calculated integral differences $dL{A}_{\alpha ,\beta }^{n}$ and $dR{A}_{\alpha ,\beta }^{n}$ between MI and background activity as

4 $$dL{A}_{\alpha ,\beta }^{n}=\mathop{\int }\limits_{t\in T}\,(L{A}_{\alpha ,\beta }^{n}(t)-B{C}_{\alpha ,\beta }^{n}(t))dt,$$

5 $$dR{A}_{\alpha ,\beta }^{n}=\mathop{\int }\limits_{t\in T}\,(R{A}_{\alpha ,\beta }^{n}(t)-B{C}_{\alpha ,\beta }^{n}(t))dt,$$

where *T* = 3 s is the trial length.

The wavelet analysis was carried out using MATLAB (R2017a; Mathworks Inc., MA, USA) and Brainstorm software.

### Cluster analysis

To perform the cluster analysis of KI and VI, we applies the hierarchical cluster analysis (HCA)^47^, a widely-used unsupervised machine learning technique. Using the HCA, we found the hierarchy in considered data in a greedy way, that allowed to uncover its structural properties, i.e., organize observed objects into subgroups.

Suppose that each participant is characterized by a point in the *M*-dimensional feature space, where *M* is a number of features necessary to describe MI of the subject. To perform HCA, we used a complete-linkage clustering which belongs to the agglomerative (“bottom-up”) group of clustering methods^47^. At the beginning of the algorithm, each participant represents its own cluster and during further iterations the existing clusters are joined into larger clusters until all points are combined into one cluster. The link between two clusters is a farthest distance in a feature space between two elements, each in its own cluster. In the mathematical form, the complete-linkage function *D*(*X*, *Y*) can be describes as

6 $$D(X,Y)=\mathop{{\rm{\max }}}\limits_{x\in X,y\in Y}d(x,y),$$

where *X* and *Y* are considered clusters, *x* and *y* are objects in *X* and *Y*, respectively, and *d*(*x*, *y*) is a distance between two objects in a feature space. This distance was calculated using Euclidean metric as

7 $$d(x,y)=\frac{1}{M}\sqrt{\sum _{i=1}^{M}\,{({x}_{i}-{y}_{i})}^{2}},$$

where *x*_*i*_ and *y*_*i*_ are an *i*-th feature of *x* and *y* objects, respectively.

Concerning the stated problem of the MI-type clustering, we considered the differences $dL{A}_{\alpha ,\beta }^{n}$ and $dR{A}_{\alpha ,\beta }^{n}$ (*i* = 1, …, *N*) as a feature set of the MI process for left and right arms, respectively. In addition, for HCA the wavelet energy differences were averaged over limbs as follows

8 $$d{E}_{\alpha }^{n}=\frac{dL{A}_{\alpha }^{n}+dR{A}_{\alpha }^{n}}{2},\,d{E}_{\beta }^{n}=\frac{dL{A}_{\beta }^{n}+dR{A}_{\beta }^{n}}{2}.$$

The dimensionality of the feature space in the case of MEG measurements is equal to 2*N* = 204 for each limb. It is clear that the number of dimensions is quite large. To reduce the dimensionality of the feature space, $d{E}_{\alpha ,\beta }^{n}$ was averaged over the channels, so that overall wavelet energy difference can be given as $d{E}_{\alpha ,\beta }=1/N\sum _{n=1}^{N}\,d{E}_{\alpha ,\beta }^{n}$. Thus, each subject is described by a point in the two-dimensional MI feature space (*dE*_*α*_, *dE*_*β*_).

We will show below that solving the MI clustering problem in a given two-dimensional feature space is more demonstrative and easy for interpretation. HCA was performed in Python using the SciPy package.

### Artificial intelligence

**Figure 2:**
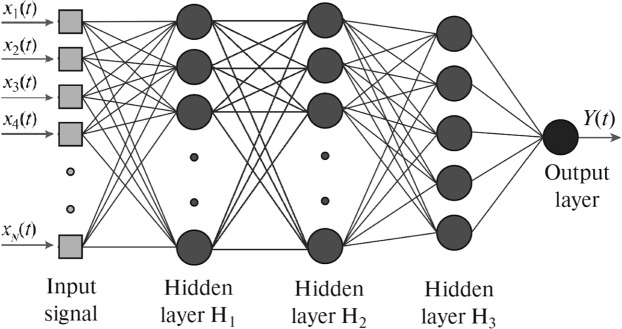
Multilayer perceptron (MLP) with input layer (IL) supplied by *N* inputs from informative MEG channels (*x*_1_, …, _*N*_), three hidden layers (H1, H2 and H3) with 30, 15 and 5 neurons, respectively, and output layer (OL) with a single neuron.

For classification of the brain states associated with MI, we used a popular type of artificial neural networks (ANN) called multilayer perceptron (MLP)^48^ schematically shown in Fig. 2. Previously, the MLP architecture was effectively used in the MEG study for detection of human decision-making uncertainty^33^ and the EEG analysis of bistable image interpretations^35^. The MLP represents a relatively simple structure of a feedforward ANN, where informative signal **X**(*t*) fed into the input layer sequentially propagates from the input layer to the output layer where the output signal *Y*(*t*) is recorded.

For the ANN analysis, we constructed the MLP which consisted of an input layer with selected number of MEG channels for training/testing the network, followed by three hidden layers with 30, 15 and 5 neurons, respectively. The output layer comprises of a single neuron. The function *Y*(*t*) describes an instantaneous brain state at time *t*_*j*_ according to the input *N*-dimensional vector signal

9 $${{\bf{X}}}_{j}(t)={({x}_{1}({t}_{j}),{x}_{2}({t}_{j}),\ldots ,{x}_{N}({t}_{j}))}^{T},$$

where *x*_*n*_(*t*_*j*_) is the event-related field from an *n*-th MEG channel measured at time *t*_*j*_ and *N* is the vector dimension equal to the number of input MEG channels taken for training/testing the neural network.

Every layer *l* in this MLP transforms the informative input signal **X**_*l*_ into

10 $${{\bf{U}}}_{l}=f({{\bf{W}}}_{l}{{\bf{X}}}_{l}+{{\bf{B}}}_{l}),$$

where ***U***_*l*_ is the output vector signal of layer *l*, **W**_*l*_ is the weight matrix of links between input elements and neurons of layer *l*, **B**_*l*_ is the vector of displacement weights, and *f* is the hyperbolic tangent sigmoid transfer function defined as

11 $$f(x)=\frac{2}{1+{e}^{-2x}}-1.$$

#### MLP teaching

We taught the MLP to classify the brain states of the neural ensemble through optimization of the weights of links and displacements by means of minimization of the root mean square error (RMSE)^48^. We used the training algorithm called *scaled conjugate gradient* because it provides higher efficiency for pattern recognition problems than other algorithms, such as the Levenberg-Marquardt. The training stopped as soon as any one of the following conditions was met, either (i) RMSE was less than 10^−5^ or (ii) the batch training with all input data ran for at least 5000 times. It should be noted that the above criteria are not universal or even the best for this particular application. They have only been found using hit-and-trial method and chosen to provide better results than the results obtained in the previous EEG studies.

#### Data filtering

The input data were filtered by a low-pass filter of order 70 with a cutoff frequency changing according to each study.

#### Data mixing

Mixing the input data usually improves the efficiency of the machine learning algorithm. In this work, we used a random mixing of the input signals corresponding to a particular task. For example, to classify MI between left and right arms, we mixed the data of all trials related to the left and right arms for each channel and defined the targets correspondingly (0 for left arm and 1 for right arm).

First, we trained the ANN using 75% of MEG trials and then tested it with the resting 25% trials. To save training time and improve accuracy, we only used 2-s trials for all subjects chosen within 1.5–3.5 s intervals of each 5-s epoch. The shorter trials did not provide sufficient information. We also tried other two strategies which could give sufficient information using shorter training time:

- Training ANN for 500 batches alternately with samples from the first and second seconds. This means that, first, we trained ANN using samples from 1.5–2.5 seconds for 500 batches, and then, we trained ANN using samples from 2.5–3.5 s for 500 batches. This constituted a single iteration. We did 500 such iterations.
- Training ANN for 500 epochs alternately with odd and even samples from the entire 2 seconds.

Changing the training method and network parameters, such as a number of input channels and cutoff frequency, we obtained interesting results about brain activity during MI.

The ANN classification was carried out in MATLAB (R2017a; Mathworks Inc., MA, USA) using the Neural Network Toolbox. Statistical significance was tested via Mann-Whitney U-test in Python using the SciPy package.

## Results

During the experimental session, each participant was sat in a comfortable chair inside the Vectorview MEG system, as shown in Fig. 1(A), and executed imagination of movements in accordance with experimental paradigm illustrated in Fig. 1(B) (see details in section “Materials and Methods”). As opposed to trained subjects instructed to perform either KI or VI^16^, the untrained subjects in our experiments were instructed to perform KI only. Some of them could manage to do KI, whereas the others resorted to VI. In order to classify the participants into VI and KI categories, we applied time-frequency and hierarchical cluster (HCA) analyses to the MEG recordings obtained during the experimental procedure.

### Event-related synchronization/desynchronization (ERS/ERD)

**Figure 3:**
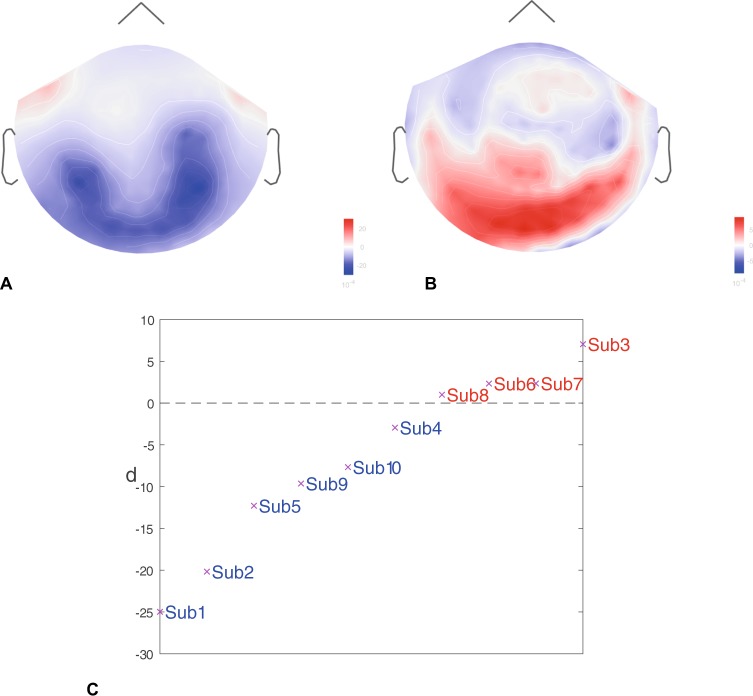
Typical results of event-related synchronizaiton/desynchronization (ERS/ERD) averaged over all trials for KI and VI. (**A**) Event-related desynchronization (ERD) in *μ*-band for KI subject 1. (**B**) Event-related synchronization (ERS) in *μ*-band for VI subject 7. (**C**) Averaged over all channels ERS/ERD degree *d* of all subjects. The subjects are classified into KI and VI groups depending on the sign of *d*, i.e., the subjects with *d* < 0 belong to the KI group, while the subjects with *d* > 0 to the VI group.

It is known, that MI is accompanied by a significant change in neural activity in *α* (8–12 Hz) and *β* (15–30 Hz) frequency bands^12,49^. Therefore, we analyzed brain dynamics in terms of ERS/ERD in these frequency bands during the MI performance, which allowed us to classify the subjects into KI and VI categories. The results are shown in Fig. 3. Figure 3(A) and 3(a) represent typical ERD and ERS distributions in *μ*-band (8–30 Hz) for KI and VI, respectively. One can see that the KI subjects (first line) have stronger ERD centralized near the inferior-parietal lobe, while the VI subjects (second line) tend to have ERS in the superior-parietal and occipital lobes. In order to quantify ERS/ERD for the time-frequency data shown above, we averaged ERS/ERD data over all 306 MEG channels to get a single value denoted as *d*. The distribution of *d* among all subjects is shown in Fig. 3(C). One can see that *d* takes negative values for KI subjects and positive for VI subjects.

### Evoked response

**Figure 4:**
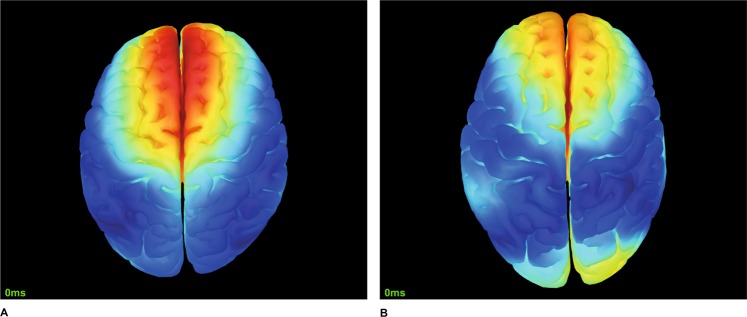
Typical evoked responses for (**A**) KI (subject 1) and (**B**) VI (subject 3). The activity in the frontal cortex for KI subjects is suppressed during MI.

The above criteria for classification into KI and VI groups was confirmed by the analysis of the evoked response, which is the average of time series over all trials. The results are present in Fig. 4, where we show the activity of the inferior part of the brain for VI (Fig. 4(A)) and KI (Fig. 4(B)) subjects (the evoked potential in the figure is averaged over 5-s trial duration). The subjects from the VI group are characterized by neural activation of the occipital cortex in contrast to the subjects from the KI group, who demonstrate activity in the premotor area, which is absent in the VI group. A possible mechanism of such a behavior will be discussed in the next section.

### Hierarchical cluster analysis (HCA)

**Figure 5:**
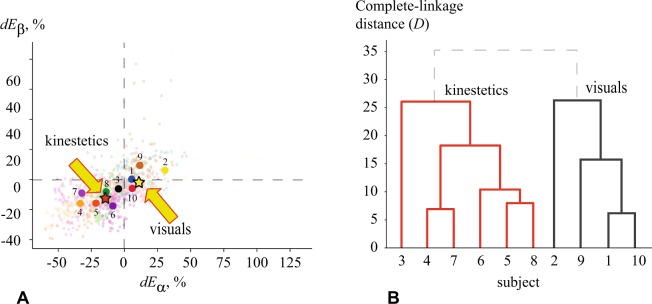
Results of hierarchical cluster analysis (HCA) illustrating the clustering of subjects belonging to KI and VI types. (**A**) Wavelet energy differences during MI in (*dE*_*α*_, *dE*_*β*_) feature space. Different colors indicate different subjects: clouds of small dots represent wavelet energy differences for *i*-th channel (*i* = 1, …, *N*) and big dots show differences in individual wavelet energy averaged over *N* = 102 channels. Stars show centroids of KI (red) and VI (yellow) clusters obtained by *k*-means clustering. (**B**) Dendrogram showing the formation of two subgroups (KI and VI) in terms of Euclidean distance between clusters in (*dE*_*α*_, *dE*_*β*_) feature space.

The HCA results are present in Fig. 5(A), where we plot coefficients $d{E}_{\alpha }^{n}$, $d{E}_{\beta }^{n}$ which characterize changes associated with MI in the event-related field in the *α* and *β* frequency bands of *n*-th MEG channel with respect to the resting state. The colored clouds of small dots show ($d{E}_{\alpha }^{n}$, $d{E}_{\beta }^{n}$) obtained from all 102 magnetometers. The dots for each subjects have different color. The dashed lines ($d{E}_{\alpha }^{n}=0$ and $d{E}_{\beta }^{n}=0$) separate the regions where the energy changes in a different way. For example, the dots above the horizontal line ($d{E}_{\beta }^{n}=0$) correspond to the MEG channels where ERS was observed in the *β*-frequency, while the dots below this line correspond to the channels where ERD took place. Similarly, the vertical dashed line separates ERS and ERD regions in the *α*-band. The big dots in Fig. 5(A) indicate the overall difference between wavelet energies in the *α* and *β* ranges for every subject, averaged over all 102 magnetometers. Therefore, each big dot can be considered as an individual MI characteristic of each participant.

The dendrogram in Fig. 5(B) shows the arrangement of clusters obtained by HCA applied to the big dots in Fig. 5(A). One can see that all subjects can be well separated into two large clusters with the exception of the upper row of the dendrogram marked by the dashed lines, i.e., subjects 3, 4, 5, 6, 7 and 8 are arranged into the KI group, while the rest of the subjects 1, 2, 9 and 10 are arranged into the VI group. It should be noted, that the links between the subjects inside each group are much smaller than the links between the clusters. This confirmes that HCA provides a good enough precision for the clustering. Comparing the dendrogram with subjects’ positions in the feature space of (*dE*_*α*_, *dE*_*β*_) (see Fig. 5(A)), one can see that the brain activity during MI of the KI subjects is characterized by well-pronounced ERD in both *α* and *β* frequency bands. Such a behavior is similar to real movement when *α*- and *β*-wave energies are suppressed in the motor brain area^50^. On the contrary, for the VI subjects the MI process is accompanied by ERS (or close to ERS) in the *α* and *β* bands, that determines a key role of imagination and self-visualization of the limb movement. Furthermore, the interviews with the participants have shown that the subjects arranged by HCA into the KI group were more prone to regular physical training, whereas the subjects belonging to the VI group were more likely to experience high cognitive load and intellectual work. Finally, using *k*-means clustering we obtained the centroids of the KI and VI groups (shown by the stars in Fig. 5(A)), the proximity to which determines the belonging of the subject to a particular cluster and consequently the type of MI. According to HCA, the untrained subjects demonstrate different scenarios in their brain activity while trying to imagine motor activity.

### Artificial neural network (ANN)

Next, we constructed a type of ANN called multilayer perceptron shown in Fig. 2, trained it and then applied it to classify MEG time series trials associated with left-arm and right-arm MI in these two groups of subjects. At the first step, we pre-processed the MEG signals (for both training and testing data) using a low-pass filter with different values of cut-off frequency *F*_*c*_. We supposed, that such type of filtration could affect dynamical properties of analysed multiple-component physiological signals^51^.

**Figure 6:**
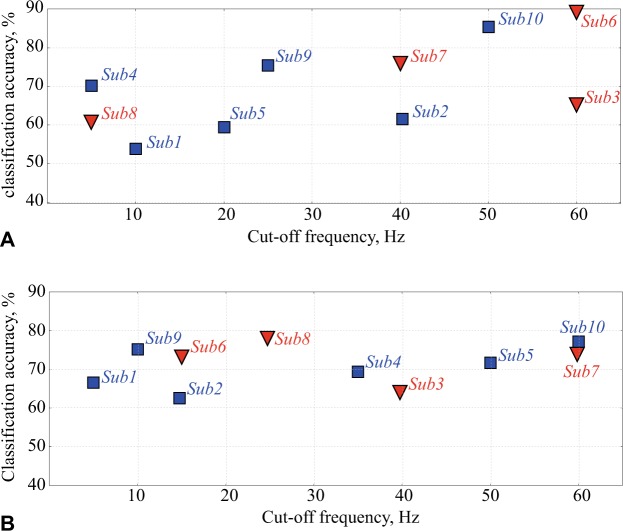
ANN classification accuracy of MI of left and right arms versus cut-off frequency for KI (squares) and VI (triangles) subjects, obtained using (**A**) 102 and (**B**) 13 channels. Each data point indicates the maximal value of the classification accuracy for every subject and the corresponding cutoff frequency *F*_*c*_, at which this maximal accuracy is achieved.

Figure 6 illustrates the variation of the maximal ANN accuracy (in %) in differentiation between MI of the left and right arms versus the cutoff frequency *F*_*c*_ of the low-pass filter for KI (squares) and VI (triangles) subjects. In Fig. 6(A) all 102 magnetometers were used for the analysis, while in Fig. 6(B) we only used 13 most informative channels localized near the left-parietal cortex. One can see that in the latter case the maximal classification accuracy almost does not change as compared with the case of using all 102 channels, and for some subjects (subjects 8 and 10) reaches 78%. However, the best accuracy is achieved by using all channels; it reaches 90% for subject 6. In both cases, the average classification accuracy over all subjects is about 70%. The obtained results demonstrate that high classification accuracy can be achieved for all subjects, regardless of which group they belong to, by the appropriate selection of the cutoff frequency of the low-pass filter.

This result is in a good agreement with the work of Graimann, *et al*.^52^, who carried out a detailed analysis of the time-frequency EEG structure and revealed that along with the motor-related brain area, MI generates relevant EEG activity in other complementary brain areas. A similar behavior has also been reported in the recent paper of Maksimenko *et al*.^50^, who found oscillatory EEG patterns associated with MI in both the low-frequency *δ*-band (<5 Hz) and motor-related *α*-band (8–12 Hz) in different brain areas simultaneously. Since the subjects demonstrated well-pronounced motor-related patterns, there was no need to increase the number of input ANN channels to extract them.

It should be noted that the results presented in Fig. 6 are closely related to the ANN optimization problem, important for classification of motor-related signals of electrical brain activity^34,53^. It is known that including all possible features of a multichannel neurophysiological data, e.g., EEG or, more significantly, MEG, results in an extremely large phase-space dimension, that has to be analyzed by the classifier. On one hand, this is a critical issue for BCI, where all calculations should be performed in real time by portable computers and the calculation performance is of extreme importance. But, on the other hand, some researchers emphasize that irrelevant EEG or MEG channels may add extra noise and redundant information that can reduce classification accuracy^54^.

## Discussion

The diverse literature reports on different brain regions involved in MI. Most data indicate major changes in the neuronal network activity of primary motor cortex (M1), posterior parietal cortex (PPC), supplementary motor area (SMA), prefrontal cortex and subcortical areas. Let us now review the most pronounced changes in different brain areas.

### Primary motor cortex (M1)

There is a discrepancy in the results of MI associated with primary motor cortex, also called M1, that can be explained to a certain extent by its definition. Many researchers previously followed the nomenclature of Talairach and Tournoux’s atlas, referring to primary motor cortex as the posterior region of the precentral gyrus, which in fact encompasses also the premotor cortex. Other scientists used the term “precentral knob” which is only a subset of M1 representing hand movements^55^. In addition, the presence of methodological differences in the experiments and the difficulty to monitor the compliance with the MI instructions^56^ only made things worse. It is not surprising that there exists a controversy in the involvement of M1 during MI with studies both in favor^7,57–60^ and against^8,61^ its involvement. Keeping the controversy aside, the studies still seem to suggest that M1 is indeed activated during MI, but much weaker as compared to real movement^6^. The role of premotor cortex (PMC) in timing the neuronal network activity is also highlighted^62,63^.

### Posterior parietal cortex (PPC)

Numerous neuroimaging studies^8,61,64–67^ indicate that PPC is actively involved in MI. When patients with lesions in PPC were asked to predict beforehand the time needed to perform movement tasks, they typically underestimated/overestimated^68^. This strongly contrasts with patients with precentral motor cortical dysfunction, who exhibit impaired movement, but retain the ability to estimate motor performance times^69^. This result suggests that there is a separate mechanism for choreographing the movement and to mentally simulate the movement for estimating the movement time, and that this time analysis mechanism is localized near the parietal cortex. It is intriguing to note that bilateral parietal lesions can cause a person to accidentally execute movements when asked to imagine them and be completely unaware about it^70^. This result hints towards the possibility that the mental MI simulation is inhibited by a mechanism also localized near the parietal lobe (in one of two hemispheres or in both) that fails in order to give way to actual movement execution during MI (see Fig. 2). Sensory modalities related to the movement can also stimulate the brain activity, such as vibratory stimuli, to induce illusion of kinesthetic activity in the primary somatosensory areas and in the M1 region^71,72^. The temporal-parietal junction has been linked with own-body imagery and self-location^73,74^. It is not surprising that KI activates motor associated areas and the inferior parietal lobe, whereas VI activates visual-related areas (occipital lobe) and the superior parietal lobe (precuneus)^75^. It should be noted, that a TMS study also reveals that the inferior parietal lobe exerts inhibitory control in the M1 region during MI^76^.

### Supplementary motor area (SMA)

Recent fMRI experiments^77^ identified SMA to be the best predictive region to distinguish between hand rotation and grasping movements which seems to suggest that the role of SMA is to translate the content of signals rather than mediating the signal. It was reported that electromyographic (EMG) activity during MI experiments, associated with failures of inhibitory control, was observed in both agnostic and antagonistic muscles as a function of a weight to be lifted in the imagination^78^ and types of muscle contraction^79^. This seems to suggest that the imagination content affects the inhibitory mechanism. Furthermore, Kasess *et al*.^80^ highlighted the contribution of supplementary motor area (SMA) in the inhibition of M1 during MI. Therefore, inhibitory mechanisms should be taken into account to explain the rest of the results.

### Prefrontal areas

Prefrontal areas, such as ventrolateral prefrontal cortex and anterior cingulate cortex, were also found to be involved in movement suppression, as well as in decision making on prioritizing the choice of movement^62,81^ (see Fig. 3).

### Subcortical areas

The results of Hanakawa^55^ show that cerebellum and basal ganglia also participate in movement suppression. In particular, the Parkinson disease affected basal ganglia causes the patients to slow down MI^82^. This indicates that basal ganglia only mediates the signal, but not affect the MI content^55^.

In their famous work, Pfurtscheller and Neuper^12^ found that mental imagery of motor actions can produce replicable EEG patterns over primary sensory and motor areas. These patterns are associated with ERD in motor-related *α*- and *β*-frequency bands, similar to those associated with motor executions^49^. At the same time, some papers report that many participants do not exhibit the expected MI related changes in their EEG^50,83,84^. According to Annett^85^, this is caused by the existence of different types of mental imagery of motor actions, namely, VI and KI modes. The knowledge of key principles of MI is needed for effective classification of EEG/MEG trials corresponding to different types of imagination and its implementation in BCI systems. It was shown^16^ that KI and VI scenarios affect the classification accuracy, e.g., the rates obtained for KI (67%) were shown to be better than the results for MI (56%).

In the present work, the conducted MEG study revealed the features of mental imagery of motor actions in untrained subject. At the first step, we analyzed imagery-related changes of event-related fields in *α*- and *β*-frequency bands in all subjects. Using the cluster analysis, we split all subjects into two groups, KI subjects demonstrating ERD in both rhythms and VI subjects demonstrating ERS in both rhythms. For both groups, we applied ANN to classify MEG trials associated with left-arm and right-arm MI. Notably, in the recent study, Grubov *et al*.^86^ could achieve classification accuracy slightly above 80% in classifying MI of hand movements using EEG data for trained subjects.

It is of curious interest to note that even though most of the brain activity is suppressed during KI in comparison to VI, we obtained higher ANN classification accuracy for the former. This can be explained by the fact that good ANN performance requires an optimum amount of data containing more task-relevant information and less noise or task-irrelevant data so that the brain activity suppression during KI enables to do this.

Finally, our results demonstrate that ANN applied for MEG time series trials for MI classification provides reasonable results for both VI and KI. However, in order to reach maximal accuracy rates, optimal input channels and appropriate filtration of input data must be used.

## Data Availability

All data are freely available from the authors on request.
